# Expression Stabilities of Candidate Reference Genes for RT-qPCR in Chinese Jujube (*Ziziphus jujuba* Mill.) under a Variety of Conditions

**DOI:** 10.1371/journal.pone.0154212

**Published:** 2016-04-26

**Authors:** Jiaodi Bu, Jin Zhao, Mengjun Liu

**Affiliations:** 1 College of Life Science, Agricultural University of Hebei, Baoding, Hebei, China; 2 Research Center of Chinese Jujube, Agricultural University of Hebei, Baoding, Hebei, China; Beijing Forestry University, CHINA

## Abstract

Reverse transcription-quantitative real-time polymerase chain reaction (RT-qPCR) is a powerful method for evaluating patterns of gene expression. Jujube whole-genome sequencing has been completed, and analysis of gene function, an important part of any follow-up study, requires the appropriate selection of reference genes. Indeed, suitable reference gene selection for RT-qPCR is critical for accurate normalization of target gene expression. In this study, the software packages geNorm and NormFinder were employed to examine the expression stabilities of nine candidate reference genes under a variety of conditions. *Actin-depolymerizing factor 1* (*ACT1*), *Histone-H3* (*His3*), and *Polyadenylate-binding protein-interacting protein* (*PAIP*) were determined to be the most stably expressed genes during five stages of fruit development and *ACT1*, *SiR-Fd*, *BTF3*, and *Tubulin alpha chain* (*TUA*) across different tissues/organs. Whereas *ACT1*, *Basic Transcription factor 3* (*BTF3*), *Glyceraldehyde-3-phosphate dehydrogenase* (*GADPH*), and *PAIP* were the most stable under dark conditions. *ACT1*, *PAIP*, *BTF3*, and *Elongation factor 1- gamma* (*EF1γ*) were the most stably expressed genes under phytoplasma infection. Among these genes, *SiR-Fd* and *PAIP* are here first reported as stable reference genes. When normalized using these most stable reference genes, the expression patterns of four target genes were found to be in accordance with physiological data, indicating that the reference genes selected in our study are suitable for use in such analyses. This study provides appropriate reference genes and corresponding primers for further RT-qPCR studies in Chinese jujube and emphasizes the importance of validating reference genes for gene expression analysis under variable experimental conditions.

## Introduction

Chinese jujube (*Ziziphus jujuba* Mill.), a member of the genus *Ziziphus* in the family Rhamnaceae, is widely distributed in temperate and subtropical areas of the Northern Hemisphere, especially the inland region of Northern China [1]. Jujube whole-genome sequencing has recently been completed [2], and gene function analysis, which is an important aspect of follow-up studies, requires the appropriate selection of reference genes. For jujube, *ZjH3* is reportedly the most suitable gene for evaluating gene expression in early-growth fruit-bearing shoots, shoot apices, and different organs by semi-quantitative reverse transcription PCR (RT-PCR) [3], and recently *UBQ*, *ACTIN9*, *UBQ2* and *CYP* were validated as stable genes at some restricted conditions by reverse transcription-quantitative real-time polymerase chain reaction (RT-qPCR) [4]. The identification and validation of additional jujube reference genes by RT-qPCR will further contribute to related studies at the transcription level.

Housekeeping genes, such as *Actin-depolymerizing factor* (*ACT*), *glyceraldehyde-3- phosphate dehydrogenase* (*GAPDH*), *α-Tubulin* (*TUA*), and *elongation factor 1-alpha* (*EF1a*), are often selected as reference genes. As stated above, ideal reference genes should exhibit stable expression levels under different experimental conditions, such as during different fruit developmental stages, in different tissues, and under various stress treatments [5–6]. However, no single gene demonstrates such constant stable expression across all conditions [7]. For example, *ACT* was found to be the least stable gene in the floral organs of pummelo [8], whereas in cherries, *TUA* and *GAPDH* were the least stable under stress treatments and different stages of fruit development [9]. Thus, to obtain accurate experimental data, it is necessary to utilize two or more reference genes, and selecting suitable reference genes and using them under appropriate conditions are critical to experimental design. Screening for suitable reference genes has been performed in many plants, including banana [10], Apricot [11], *Pyrus pyrifolia* [12], litchi [13], *Vitis vinifera* [14], and citrus [15].

Although RT-qPCR is a precise technique widely used for gene expression analysis [16–17], many factors, such as PCR conditions, RNA quality, RNA stability, and retrotranscription efficiency, can influence the reliability of the results [16, 18]. To avoid invalid results and minimize technical variations, it is necessary to validate the expression stability of selected reference genes under various conditions [16]. Genes with minimal expression variation are considered to be ideal reference genes.

In the present study, *Z*. *jujuba* Mill. ‘Dongzao’ was used as the experimental material, and nine candidate reference genes were assessed by RT-qPCR under variable conditions and treatments. The expression stability of these genes was evaluated using geNorm [19] and NormFinder [20], and their suitability as reference genes was then confirmed by RT-qPCR analysis.

## Materials and Methods

### Ethics statement

Twenty-year-old *Z*. *jujuba* Mill. ‘Dongzao’ trees were used in this study. The field samples were collected from the National Jujube Germplasm Resources Nursery (NJGRN) located in Taigu County, Shanxi Province, China. No specific permission was required for sample collection at this location; NJGRN is supported by the Chinese government as an open platform for jujube fundamental research. We confirm that the field studies did not involve endangered or protected species.

### Plant materials

#### Different tissues or organs treatment

Seven organs/tissues, including root, leaves, flowers, fruits, buds, young stems, and old stems were harvested from three trees of ‘Dongzao’ in 2015 and each treatment was repeated three times.

#### Five fruit-ripening stages treatment

There are five critical stages in jujube fruit development: young fruit (Y), pre-white ripening stage (PW), white ripening stage (W), half-red ripening stage (HR), and whole-red ripening stage (WR). Fruit samples of ‘Dongzao’ were collected at these five stages and each treatment was harvested from three trees.

#### Phytoplasma infection treatment

Jujube witches’ broom disease (JWB), caused by a phytoplasma, is one of the most severe diseases in jujube production [21]. Three diseased trees of ‘Dongzao’ about 15-year-old used in this study were from the Experimental Station of Chinese Jujube, Agricultural University of Hebei (AUH), in Baoding, Hebei. Four tissues representing different degrees of disease severity, i.e., apparent normal leaves (ANL), phyllody leaves (P), witches’-broom leaves (WBL), and healthy leaves (HL) were collected from the three trees and each treatment was repeated three times.

#### Dark treatment

Jujube seedlings of ‘Dongzao’ were cultured in Research Center of Chinese Jujube, AUH and transferred to an opaque box. Leaf samples were collected at 0, 4, 8, 12, and 16 days post-treatment.

Above collected samples were frozen directly in liquid nitrogen and then stored at -80°C until use.

### Total RNA extraction and first-strand cDNA synthesis

Total RNA was extracted using the modified CTAB method [22], and genomic DNA was removed by RNase-free DNase I (Tiangen). The quantity and quality of the RNA were determined using a NanoDrop2000. The OD_260/280_ value for all RNA samples was from 1.9 to 2.0, and the OD_260/230_ value was approximately 2.0, indicating high-purity RNA. The RNA integrity was assessed by electrophoresis through 1% (w/v) agarose gels.

First-strand cDNA was synthesized by reverse transcribing 500 ng of total RNA with PrimeScriptHRT reagent Kit (Perfect Real Time) (TaKaRa, Japan). All cDNA samples were stored at -20°C until use.

### Primer design and RT-qPCR

Based on the genome sequencing of jujube and its transcriptional data of different tissues/organs [2], nine candidate reference genes were selected. Primers for the nine tested genes were designed using Primer Premier 5.0. RT-qPCR was performed with a Bio-Rad iQ™5 using TransStart Top Green qPCR SuperMix AQ131 (TransGen Biotech, China). The primers were synthesized by Shanghai Biological Engineering Technology Services Company. The 20 μL reaction system contained 10 μL 2×SYBR Premix Ex Taq™, 0.4 μL 10 μM primers, 8.2 μL ddH_2_O and 1 μL diluted cDNA. The thermal profile for RT-qPCR was preincubation for 30 s at 94°C, followed by 40 cycles of 5 s at 94°C, 15 s at 54°C and 15 s at 72°C. Each amplification was repeated in triplicate. Primer specificity was determined by RT-qPCR and melting-curve analysis; the specificity of the amplicons was confirmed by the presence of a single peak.

### Analyses of gene stability and number of optimal reference genes

The Ct value of each reference gene was used to compare expression levels among different jujube samples. Raw Ct values were transformed into the relative quantities required as data input for geNorm and NormFinder based on the formula Q = 2^-△Ct^ The Q value was imported into geNorm 3.5 and NormFinder for reference gene selection. These algorithms rank reference genes according to the calculated gene expression stability value (M value) and an average pairwise variation of the template normalization factor (V_n_/V_n+1_). The default value suggested by geNorm is M = 1.5 [23–24], and the most stable genes have the lowest M values [25]. With a pairwise variation (V_n_/V_n+1_) ≤ 0.15, it is not necessary to introduce additional reference genes [26]. NormFinder generates a similar measure of gene expression stability (M value) through analysis of variance and the direct assessment of genetic stability [5].

A standard curve was produced using RT-qPCR data. Purified PCR product was used as the starting template, followed by five 10-fold serial dilutions, generating a gradient of concentrations (the serial dilution ranged from 10^0^ to 10^5^). The amplification efficiency of each primer pair was calculated (E = (10^−1/slope^ -1) ×100).

## Results

### Primer specificity and efficiency

Details of the nine genes and the primer pairs designed in this study are listed in Table 1. The specificity of PCR amplification for each primer pair was supported by melting curve analysis and verified by examination of the product on a 2% agarose gel. All melting curves of primer pairs showed a single peak (S1 Fig); a single PCR amplification product of the expected size for each candidate gene was detected, whereas non-specific amplification products were not observed (S2 Fig). These results proved that the primer pairs are highly specific. A standard curve was also generated according to the results of RT-qPCR (S3 Fig).

**Table 1:** Primer sequences and related information for each candidate reference gene.

Gene symbol	Gene name	Accession Number	Primer sequence (5’-3’)	Size (bp)
*EF1α*	Elongation factor 1-alpha	KT381862	F-TTCGTCTCCCACTTCAGGAT R-GGGCAAAGGTCACAACCATA	108bp
*PAIP*	Polyadenylate-binding protein-interacting protein	KT381858	F-CTTGGGAACCCTGAGAA R-GTGCCGTAAGAACCATAGA	134bp
*His3*	Histon-H3	KT381861	F-TCGCTCAGGATTTCAAGAC R-GAACAGACCGACCAAGTAA	92bp
*BTF3*	Basic Transcription factor 3	KT381863	F-TACTGGTGGGAAGGGTAGCAR-TGGAGGCTTGAACTTTAGGG	188bp
*ACT1*	Actin-depolymerizing factor1	KT381859	F-AGCCTTCCTGCCAACGAGT R-TTGCTTCTCACCCTTGATGC	125bp
*EF1γ*	Elongation factor 1-gamma	KT381860	F-TCGCTGGAGATTGATGCTAA R-CAAGATGCAGGTTCAAAGCA	133bp
*GAPDH*	Glyceraldehyde-3-phosphate dehydrogenase	KP147910	F-CAGGAACCCAGAGGAGAT R-CCACCCTTTAGATGAGCAG	108bp
*TUA*	Tubulin alpha chain	KT381863	F-CACCCACCGTTGTTCC R-TCCTTCCTCCATACCCTC	173bp
*SiR-Fd*	Sulfite reductase [ferredoxin]	KT381856	F-GCTATCAGATTTGGCTTGGA R-GGCTCTTTAGATTGCCGTTT	142bp

The accuracy of RT-qPCR data requires that all primer pairs used should have similar amplification efficiency, usually between 90–110% [27]. The PCR efficiency (E) of the primer pairs used in this study was from 92.9 to 107.5% (Table 2), indicating that the conditions are optimal and that the results obtained should be highly repeatable. The regression coefficient (R^2^) (Table 2) was larger than 0.99, indicating that the primer pairs are highly efficient and specific and thus can be used in further experiments.

**Table 2:** Efficiency of designed primer pairs of 9 candidate genes used for RT-qPCR amplification.

Gene name	Tm°C	PCR efficiency (%)	Regression coefficient (R^2^)
*EF1α*	83.0	105.7	0.995
*PAIP*	83.0	92.9	0.996
*His3*	86.0	106.4	0.997
*BTF3*	82.0	93.2	0.996
*ACT1*	82.5	107.5	0.992
*EF1γ*	84.5	100.3	0.998
*GAPDH*	84.5	102.3	0.998
*TUA*	85.0	99.0	0.997
*SiR-Fd*	80.5	103.0	0.998

### Expression stability of nine candidate reference genes

**Fig 1:**
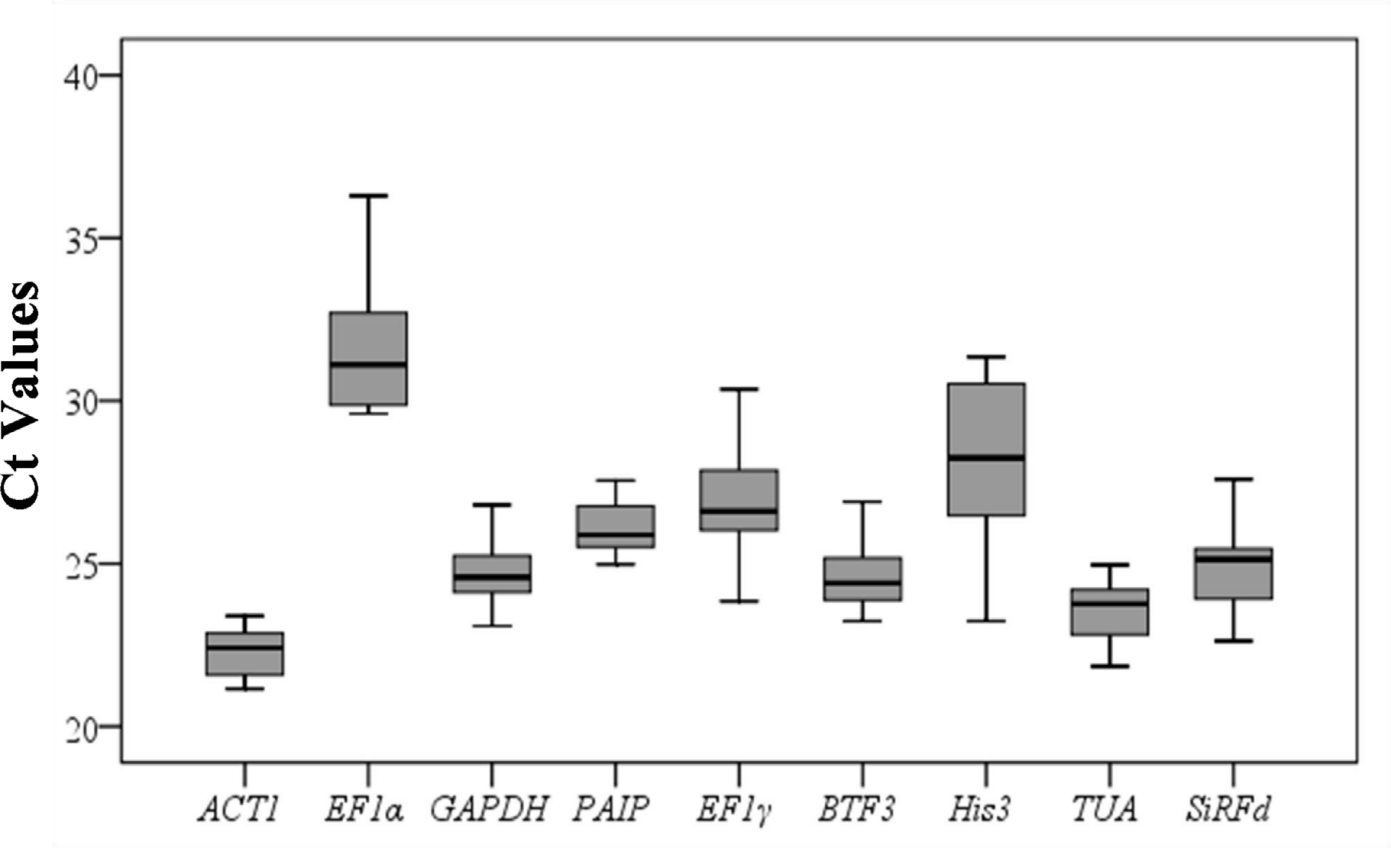
Ct values for nine candidate reference genes in all samples. The final Ct value of each sample was the mean of three biological and technical replicates. The boxes represent the 25th and 75th percentiles of data. A line across the box showed as the median. Whiskers represent the maximum and minimum values.

Cycle threshold (Ct) analysis Variation in cycle threshold (Ct) values under different conditions could reflect the stability of genes to a certain extent. By calculating the Ct value, we can distinguish the gene expression level of each candidate reference gene, whereby differences in Ct values (coefficient of variation) indicate the expression stability of that reference gene. In this study, the Ct values of the nine tested genes were collected under different experimental conditions (Fig 1), and the results presented a relatively wide range, from 21.16 for *ACT1* to 36.30 for *EF1α*. The expression levels of *ACT1* were between 21.16 and 23.40, higher than the other eight genes, indicating that *ACT1* is the most stably expressed gene in all jujube tissues examined.

#### geNorm analysis

**Fig 2:**
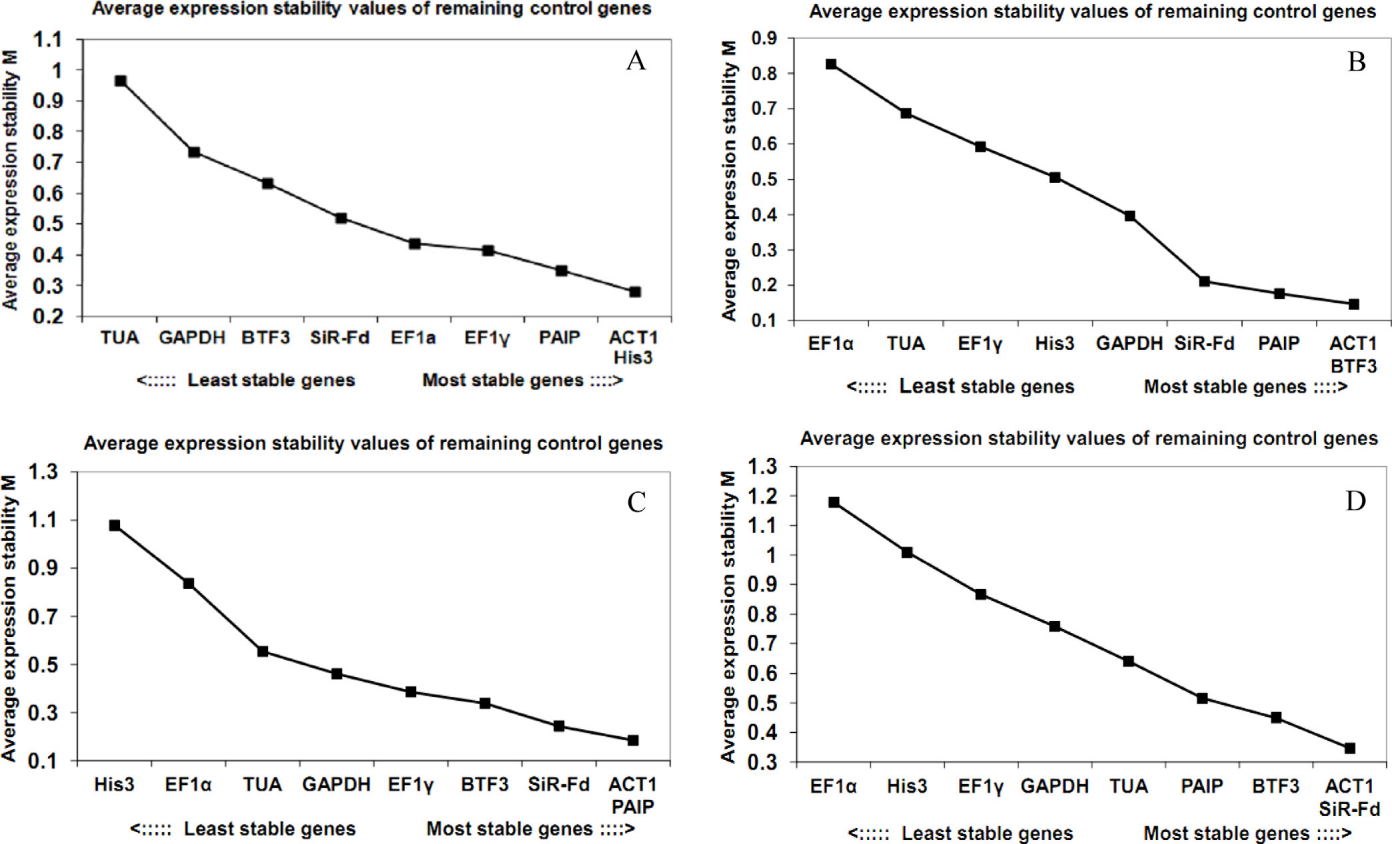
Average expression stability values (M values) calculated by geNorm. (A) Different fruit ripening stages, (B) Dark treatment, (C) Phytoplasma infection, (D) Different tissue/organs.

geNorm analysis ranks reference genes according to their expression stability (M value), and a candidate gene with an M value ≤1.5 is considered to be a viable reference gene. The M values for the nine tested genes in our study were all lower than 1.5 (Fig 2).

*ACT1* and *His3* were the most stably expressed genes in five critical fruit development stages, with M values of 0.28; in contrast, *TUA* showed the highest M value (0.97), indicating the worst expression stability among the nine tested genes (Fig 2A). *ACT1* and *BTF3* were the most stably expressed genes under dark conditions, with M values of 0.15; *EF1α*, with the highest M value of 0.82, was the least stably expressed gene (Fig 2B). *ACT1* and *PAIP* were the most stably expressed under phytoplasma infection (Fig 2C), and *ACT1* and *SiR-Fd* were the most stably expressed genes under different tissues/organs (Fig 2D). Overall, geNorm analysis revealed *ACT1* as the most stably expressed gene under all conditions, which is consistent with the Ct value results shown in Fig 1.

**Fig 3:**
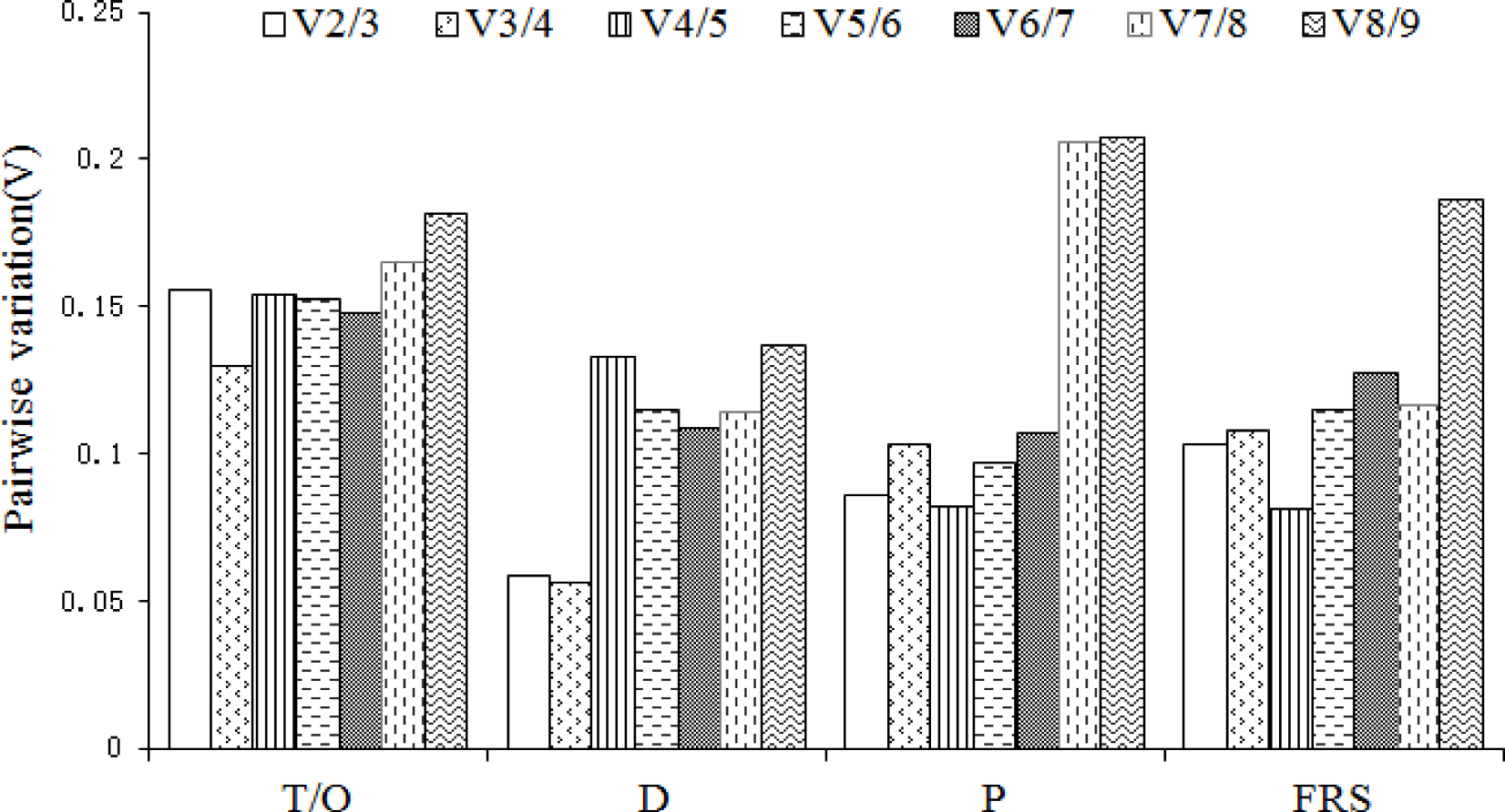
Pairwise variation (V) calculated by geNorm to determine the optimal number of reference genes. The average pairwise variations V_n_/V_n+1_ was analyzed between the normalization factors NF_n_ and NF_n+1_ to indicate the optimal number of reference genes required for RT-qPCR data normalization in different samples. T/O: Different tissue/organs, D: Dark conditions, P: Phytoplasma infection, FRS: Fruit ripening stages.

To obtain reliable results from RT-qPCR studies, two or more reference genes should be used for data normalization. By employing geNorm software, we analyzed the pairwise variation (V_n/n+1_) to determine the optimal number of genes required for normalization. According to the results (Fig 3), V_2/3_ were lower than 0.15 under most experimental conditions, indicating two stable reference genes as the optimal number, except for tissues/organs, in this study; three reference genes (V_3/4_ < 0.15) would be needed for normalizing gene expression in different tissue/organs. It should be noted that a greater number of reference genes does not necessarily mean more reliable results.

#### NormFinder analysis

NormFinder software is another tool used for evaluating the stability of reference genes. More stably expressed genes have lower M values. Based on the results of NormFinder (Table 3), the most stably expressed genes at the fruit-ripening stages were *PAIP* and *His3*. *BTF3* and *TUA* were the most stable across different tissues/organs, *GADPH* and *PAIP* under dark conditions, *BTF3* and *EF1γ* under phytoplasma infection. These results were not consistent with those of geNorm.

**Table 3:** Ranking of candidate reference genes in order of M value as calculated by NormFinder.

Rank	Fruit riping stages	Tissue/organs	Dark treament	Phytoplasma infection
1	*PAIP* (0.121)	*BTF3*(0.145)	*GAPDH*(0.116)	*BTF3*(0.121)
2	*His3*(0.250)	*TUA*(0.262)	*PAIP* (0.300)	*EF1γ* (0.121)
3	*ACT1*(0.276)	*PAIP* (0.43)	*His3*(0.326)	*GAPDH*(0.123)
4	*EF1γ* (0.311)	*SiR-Fd* (0.503)	*BTF3*(0.345)	*TUA*(0.286)
5	*EF1a* (0.473)	*ACT1*(0.504)	*ACT1*(0.355)	*ACT1*(0.304)
6	*GAPDH*(0.476)	*GAPDH*(0.557)	*EF1γ* (0.362)	*PAIP* (0.351)
7	*BTF3*(0.486)	*EF1γ* (0.62)	*SiR-Fd* (0.485)	*SiR-Fd* (0.409)
8	*SiR-Fd* (0.504)	*His3*(0.862)	*TUA*(0.549)	*EF1a* (1.220)
9	*TUA* (1.198)	*EF1a*(1.121)	*EF1a* (0.842)	*His3*(1.279)

### Validation of selected reference genes by RT-qPCR

**Fig 4:**
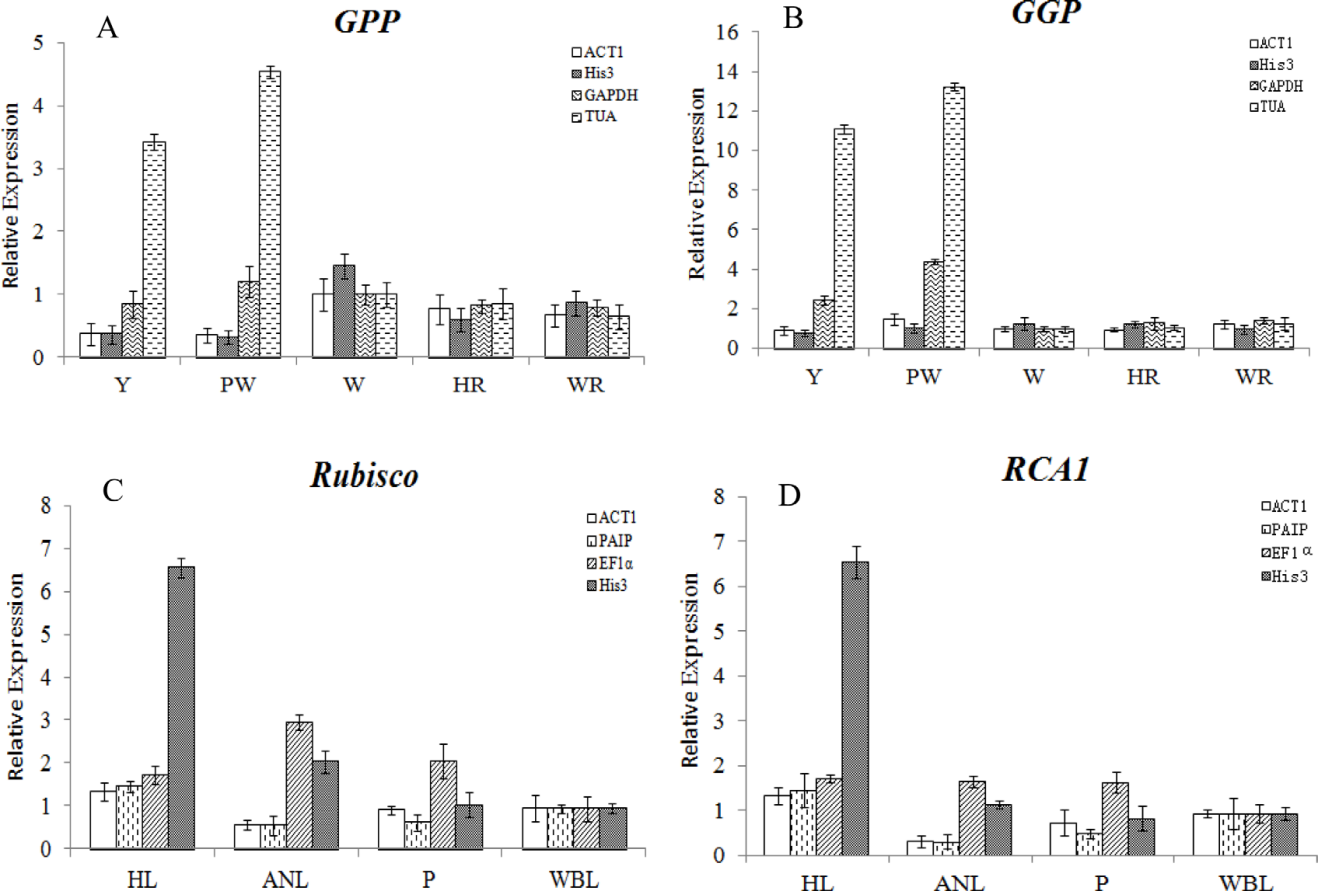
Relative expression using validated reference genes for normalization under different experimental conditions. (A) and (B): Relative expression of *GPP* and *GGP* in five fruit ripening stages, normalized by *ACT1*, *His3*, *GAPDH*, or *TUA*; Y: young fruit, PW: pre-white ripening stage, W: white ripening stage, HR: half-red ripening stage, WR: whole-red ripening stage. (C) and (D): Relative expression of *Rubisco* and *RCA1* under phytoplasma infection, normalized by *ACT1*, *PAIP*, *EF1α*, or *His3*. HL: healthy leaves, ANL: apparent normal leaves, P: phyllody leaves, WBL: witches’ broom leaves.

To demonstrate the importance of selected reference genes, the expression of two functional genes involved in L-ascorbic acid (AsA) biosynthesis, GDP-L-galactose phosphorylase (*ZjGGP*) and L-galactose-1-P phosphatase (*ZjGPP*), were evaluated by RT-qPCR at five fruit-ripening stages (Fig 4A and 4B), and the expression of two key genes related to photosynthesis, Ribulose-1,5-bisphosphate carboxylase/oxygenase (*ZjRubisco*) and Rubisco activase 1 (*ZjRCA1*), were evaluated under phytoplasma infection (Fig 4C and 4D).

As shown in Fig 4A, *ZjGPP* expression was highest at the white ripening stage (W) when using *ACT1* and *His3* as the internal reference genes, while its expression at the young stage (Y) and pre-white ripening stage (PW) was higher than other stages when using *GADPH* and *TUA* as the reference genes. Such differences between stable and unstable reference genes were also found with regard to *ZjGGP* expression (Fig 4B). The results proved that when normalized with stable reference genes, the expression patterns of *ZjGPP* and *ZjGGP* were in accordance with physiological data, i.e., AsA content of jujube fruit at the white ripening stage was higher than at the other stages.

For calculating jujube gene expression during phytoplasma infection, four tissues representing different degrees of disease severity were used for the selection of appropriate reference genes. The two typical symptoms, phyllody and witches’ broom directly result in an absence of fruit output in diseased jujube trees. After phytoplasma infection, the apparent normal leaves (ANL), phyllody leaves (P), and witches’-broom leaves (WBL) from diseased jujube trees turned yellow to some degree, indicating that their photosynthetic capacity was decreased compared to that of healthy leaves. The samples were collected on August 5^th^, 2015; the phyllody (P) and witches’-broom (WBL) leaves were younger than the apparent normal ones (ANL), and the chlorophyll contents of P and WBL leaves were higher than those of ANL ones and lower than those of healthy ones (data not shown). Thus, the expression pattern of *Rubisco* and *RCA1* normalized using *ACT1* and *PAIP* as internal reference genes (Fig 4C and 4D) showed good consistency with the observed phenotypes. However, the expression pattern was dramatically altered when normalized using unstable references (*EF1a* and *His3*). Moreover, the expression patterns of the target genes normalized to the two selected stable references were in accordance with each other.

## Discussion

At present, RT-qPCR is widely applied for assaying gene expression in plant cells. “Determine which reference genes are best for normalization of test gene transcript levels amongst all samples” is considered one of eleven golden rules of RT-qPCR [16], and the accuracy of results is strongly influenced by the stability of the internal reference genes used for data normalization. In this study, the expression stabilities of nine candidate genes, *ACT1*, *TUA*, *BTF3*, *GAPDH*, *SiR-Fd*, *EF1α*, *EF1γ*, *His3*, and *PAIP*, were evaluated under various conditions by geNorm and NormFinder. *ACT1*, *GAPDH* and *His3*, which are often used as internal controls for expression analyses [6, 7, 28], were found to be stable reference genes under some conditions in our study. In addition, the new stable reference genes *SiR-Fd* and *PAIP* have not been reported in previous research. In this study, *PAIP* was the most stable reference gene under phytoplasma infection conditions and the second most stable gene during five fruit-ripening stages and dark conditions according to geNorm analysis. These new reference genes will enrich the pool of reference genes, and indicating that additional stable reference genes should be identified by screening.

NormFinder and geNorm were both used to evaluate the stability of reference genes, though the results were not very consistent under some experimental conditions as a result of the different statistical methods used. Under phytoplasma infection, *ACT1* and *PAIP* were identified as suitable reference genes by geNorm, whereas *BTF3* and *EF1γ* were identified as suitable ones by NormFinder. Differences between the two programs were also revealed when examining longan [29], *Pyrus pyrifolia* [12], and rubber trees [30]. *ACT1* performed well under all experimental conditions and was determined to be suitable for use as an internal control gene in Chinese jujube, as in other species [10, 13, 31].

In previous studies of Chinese jujube, *ZjH3* was reported as the most suitable housekeeping gene for evaluating jujube fruit-bearing shoot development by semi-quantitative RT-PCR [3], whereas, its expression was not the most stable at different fruit developmental stages, tissues and genotypes by RT-qPCR[4]. In the present study, the expression of *ZjH3* was relatively stable only at fruit developmental stages. Above difference should be caused by the different genes, tissues and conditions tested. In addition, we found that the M values of most of tested genes in our study and in Zhang *et al*. [4] were lower than 1.5, meaning that these tested genes had stable expression levels and could be applied as reference genes under some specified conditions. Thus, the screening result was significantly influenced by the genes evaluated at the same time and the genes selected were relatively stable compared to others. The reference genes selected in present study and previous studies [3, 4] provide more choices for further molecular mechanism studies in Chinese jujube. When normalizing with the most stable reference genes, we should comprehensively consider additional factors and employ multiple programs.

## Conclusions

In this study, suitable reference genes for RT-qPCR in Chinese jujube were evaluated under a variety of conditions by the software packages geNorm and NormFinder. We have identified *ACT1*, *His3*, and *PAIP* as suitable reference genes for fruit development, and *ACT1*, *SiR-Fd*, *BTF3*, and *TUA* across different tissues/organs. *ACT1*, *BTF3*, *GADPH*, and *PAIP* were the most stably expressed genes under dark conditions. *ACT1*, *PAIP*, *BTF3*, and *EF1γ* were the most stable ones under phytoplasma infection. Overall, *ACT1* was an ideal reference gene under all above conditions. Moreover, two novel reference genes, *SiR-Fd* and *PAIP*, were first reported. The reference genes selected in present study provide more choices for further gene expression analysis and functional studies in Chinese jujube.

## Supporting Information

S1 Fig Melting curves for the nine candidate reference genes. (TIF)

S2 Fig RT-qPCR amplification specificity of the nine candidate reference genes. Amplification fragments were separated by 2% agarose gel electrophoresis.
(TIF)

S3 Fig RT-qPCR standard curve of the nine candidate reference genes. (TIF)
