# Supplementary figures and images for: Expression Stabilities of Candidate Reference Genes for RT-qPCR in Chinese Jujube (Ziziphus jujuba Mill.) under a Variety of Conditions
Source: PLoS One. 2016 Apr 26;11(4):e0154212. doi: 10.1371/journal.pone.0154212 (PMC4846040; doi:10.1371/journal.pone.0154212)

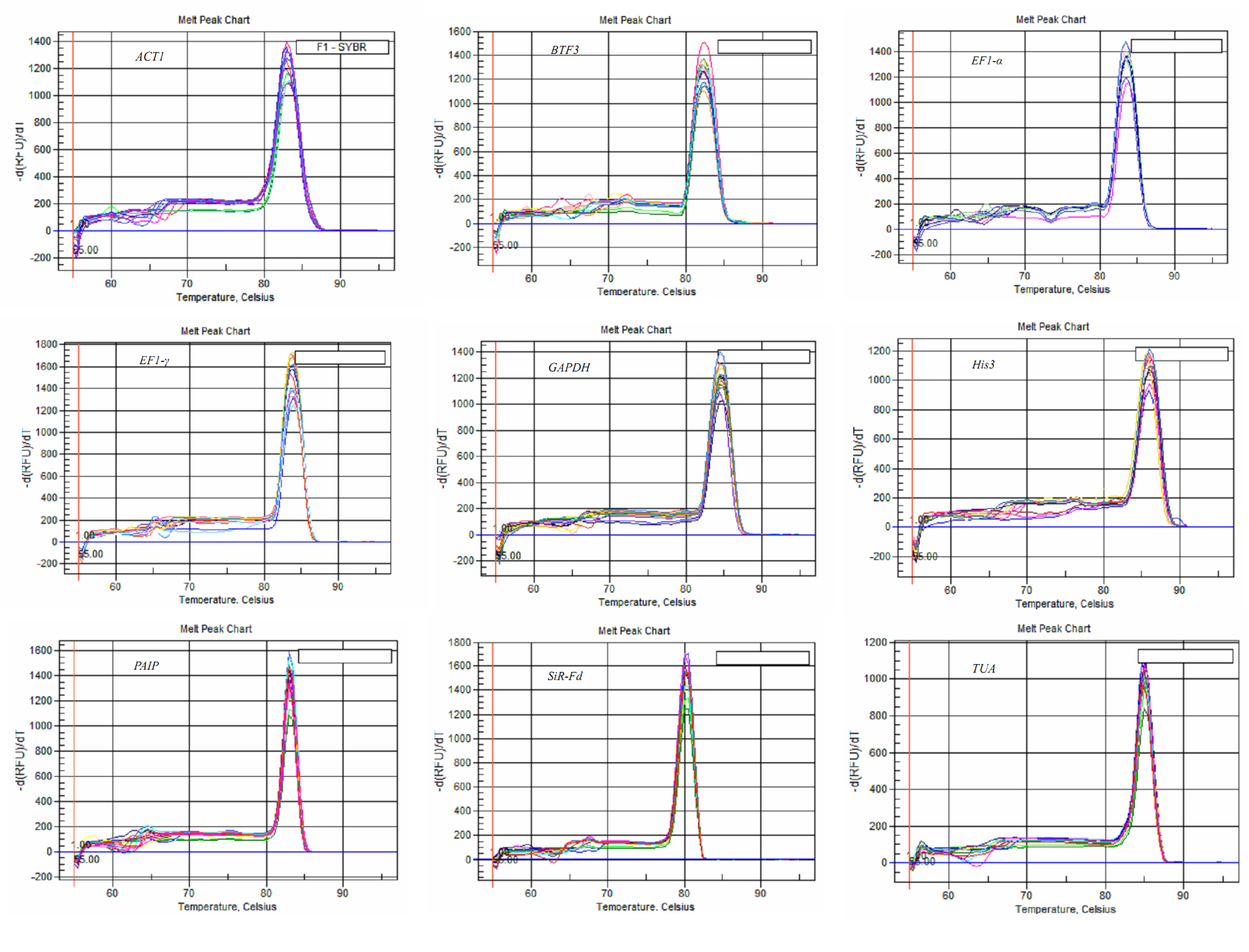

Supplement: S1 Fig — (TIF) [file pone.0154212.s001.tif]

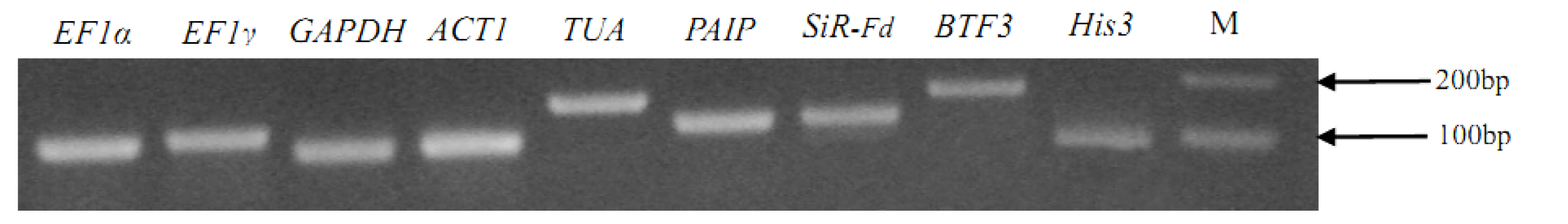

Supplement: S2 Fig — Amplification fragments were separated by 2% agarose gel electrophoresis. (TIF) [file pone.0154212.s002.tif]

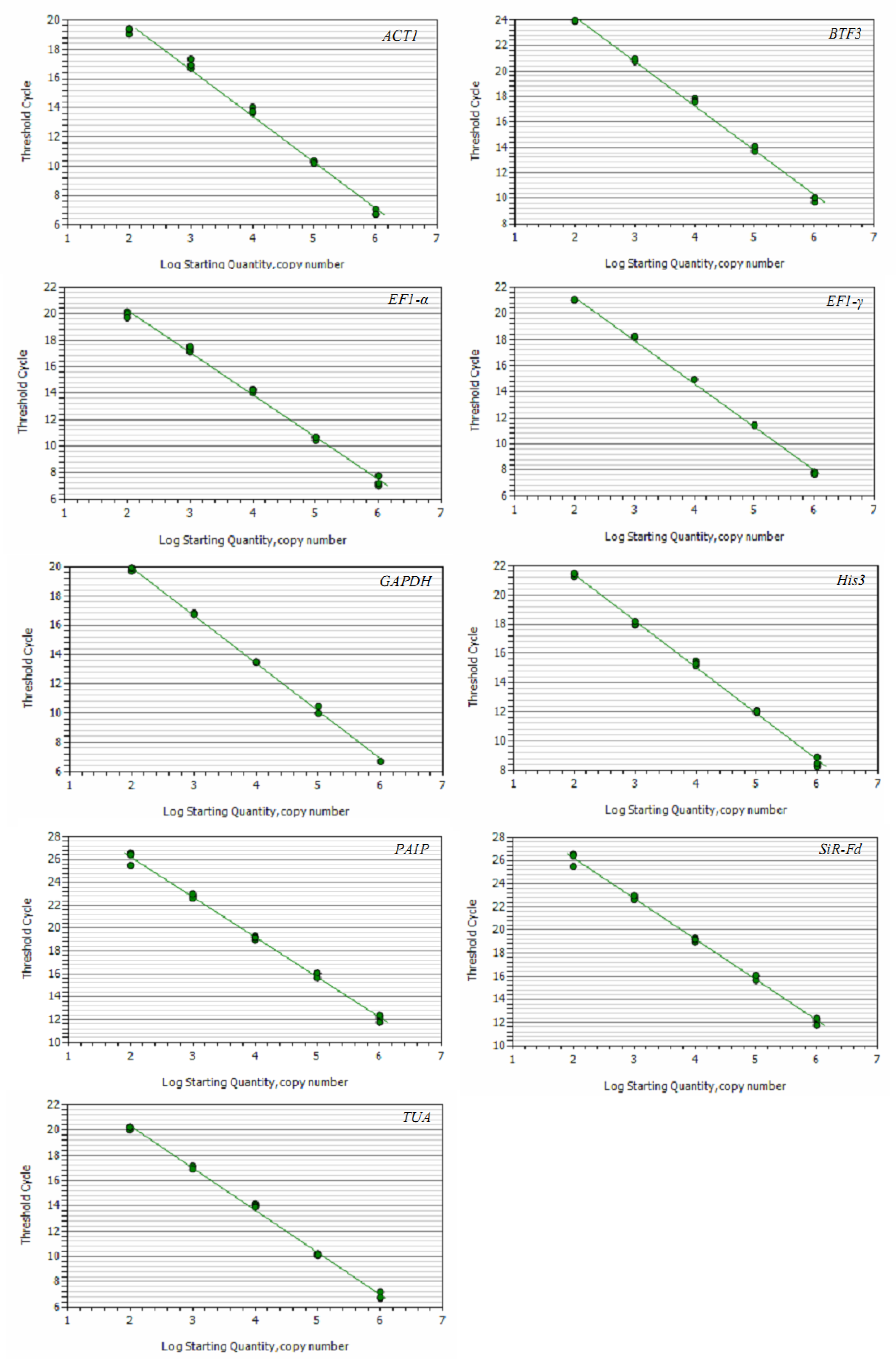

Supplement: S3 Fig — (TIF) [file pone.0154212.s003.tif]
